# Pleural Effusion in a Patient With Splenic Hematoma Following Sleeve Gastrectomy: A Case Report

**DOI:** 10.7759/cureus.108048

**Published:** 2026-04-30

**Authors:** Sara Abu-Ghazal, Ahmad Alromi

**Affiliations:** 1 Department of General Surgery, New Zarqa Governmental Hospital, Zarqa, JOR; 2 Department of General Surgery, Al Bashir Hospital, Amman, JOR

**Keywords:** bariatric surgery, pleural effusion, rare complication, sleeve gastrectomy, splenic hematoma

## Abstract

Sleeve gastrectomy has proved both effective and durable as a surgical intervention for obesity worldwide. This emphasizes the importance of reporting uncommon complications to enhance surgeons’ awareness in their prevention, recognition, and management. We report an unusual case of a 31-year-old female with a BMI of 46 kg/m² who presented with progressive left hypochondrial pain and leukocytosis two weeks after laparoscopic sleeve gastrectomy. Abdominal CT revealed a splenic hematoma with associated left-sided pleural effusion. Surgical intervention was required for pleural effusion management after unsuccessful conservative treatment while ensuring splenic preservation. This case highlights splenic hematoma following sleeve gastrectomy as an uncommon but important cause of pleural effusion.

## Introduction

Laparoscopic sleeve gastrectomy has emerged as a highly effective metabolic and bariatric surgery for the management of morbid obesity. It is considered a safe procedure with a low rate of morbidity and mortality. Hemorrhage, anastomotic leak, and intra-abdominal abscess are among the frequently reported early postoperative complications, whereas gastric outlet stenosis, nutritional deficits, and gastroesophageal reflux disease are common late postoperative complications [1].

While the majority of the literature focuses on the more common complications, the less common complications remain underreported. Rare complications after laparoscopic sleeve gastrectomy include various fistulas, splenic injury, solid organ abscesses, esophageal perforation, pancreatic leak, and vascular events [2].

Splenic injury during laparoscopic sleeve gastrectomy is relatively uncommon but can lead to significant morbidity. Capsular tears, hematomas, ischemia, abscesses, ruptures, and vascular damage were among the documented events [3-5]. Meanwhile, thoracic complications post bariatric surgery are underreported [6]; left-sided pleural effusion resulting from splenic hematoma after sleeve gastrectomy is an exceedingly rare and serious complication. This case highlights splenic hematoma following sleeve gastrectomy as an unexpected etiology of pleural effusion. The correlation could be explained by hematoma-induced diaphragmatic irritation that results in reactive pleural inflammation.

## Case presentation

**Figure 1:**
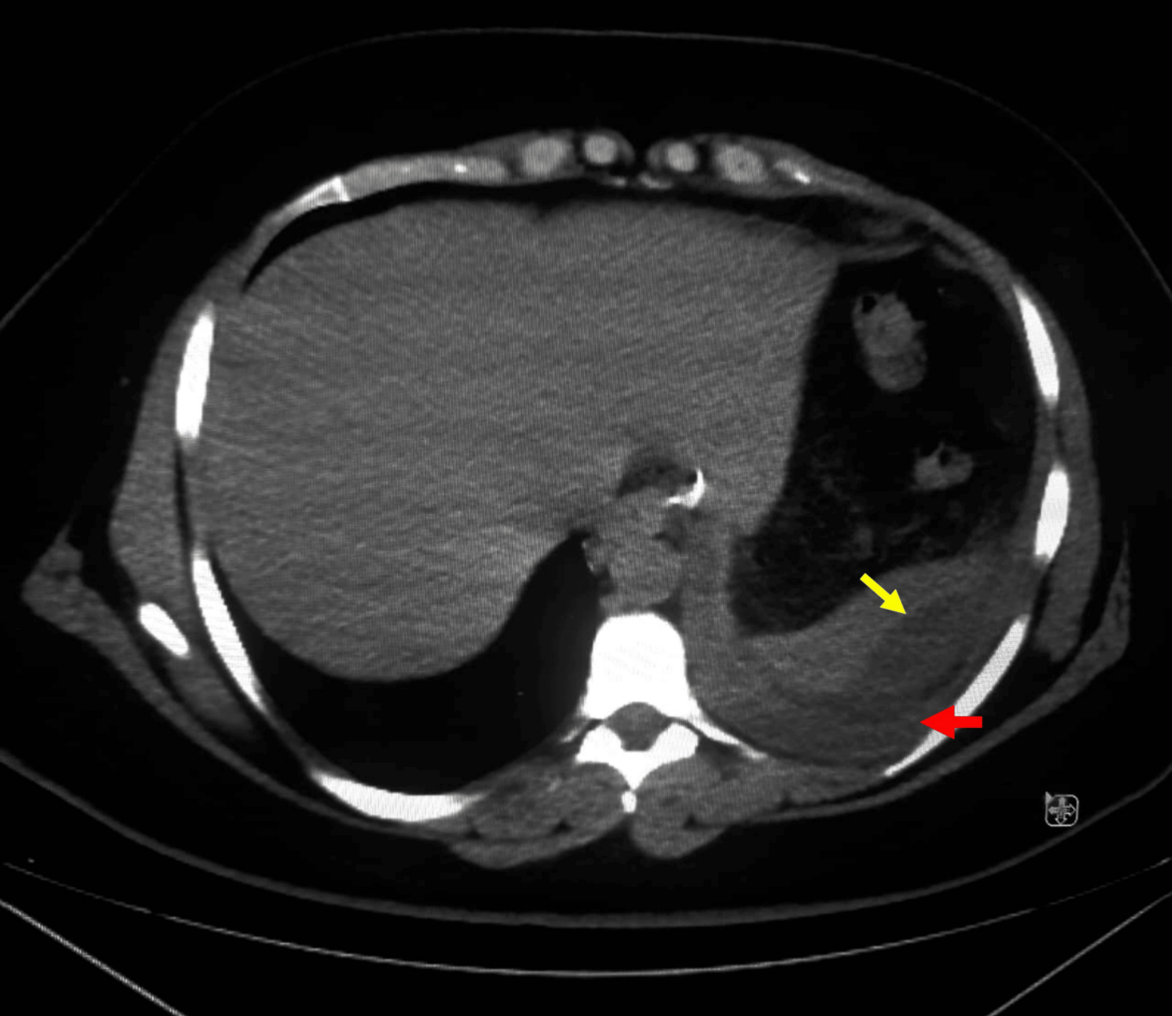
Contrast-enhanced abdominal CT in the axial plane taken two weeks after laparoscopic sleeve gastrectomy showed a splenic hematoma (yellow arrow) accompanied by a mild left-sided pleural effusion (red arrow).

A 31-year-old female with a BMI of 46 kg/m² underwent laparoscopic sleeve gastrectomy. She had an uneventful recovery and was discharged on postoperative day one. Two weeks later, the patient arrived at the emergency department reporting abdominal pain that started two days prior. The pain started progressively in the left hypochondrium, accompanied by nausea and vomiting. Initially, it was not associated with any respiratory symptoms. The vital signs were within normal limits, and the physical examination was unremarkable, other than left hypochondrium tenderness. Blood tests revealed a stable hemoglobin level and an increased white blood cell count of 17.7 × 10⁹/L (reference range: 4.0-11.0 × 10⁹/L). Blood cultures remained negative throughout the hospital stay. A contrast-enhanced abdominal CT scan was performed to rule out a staple-line leak, given its position as the leading differential diagnosis. Unexpectedly, the imaging instead revealed a splenic hematoma accompanied by a mild left-sided pleural effusion (Figure 1).

The splenic hematoma was managed conservatively, given its small size and the patient’s hemodynamic stability. As the pleural effusion was asymptomatic, supportive management was pursued, and empirical antibiotic therapy was initiated as a precautionary measure.

**Figure 2:**
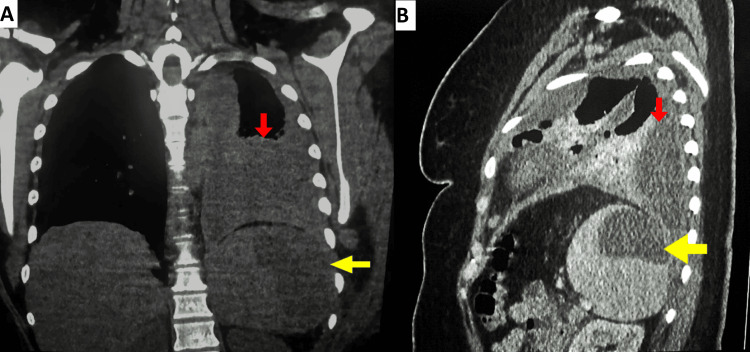
Contrast-enhanced CT in the coronal plane (A) and sagittal plane (B) revealed an expansion of the splenic hematoma (yellow arrows) and an increase in the left-sided pleural effusion (red arrows).

**Figure 3:**
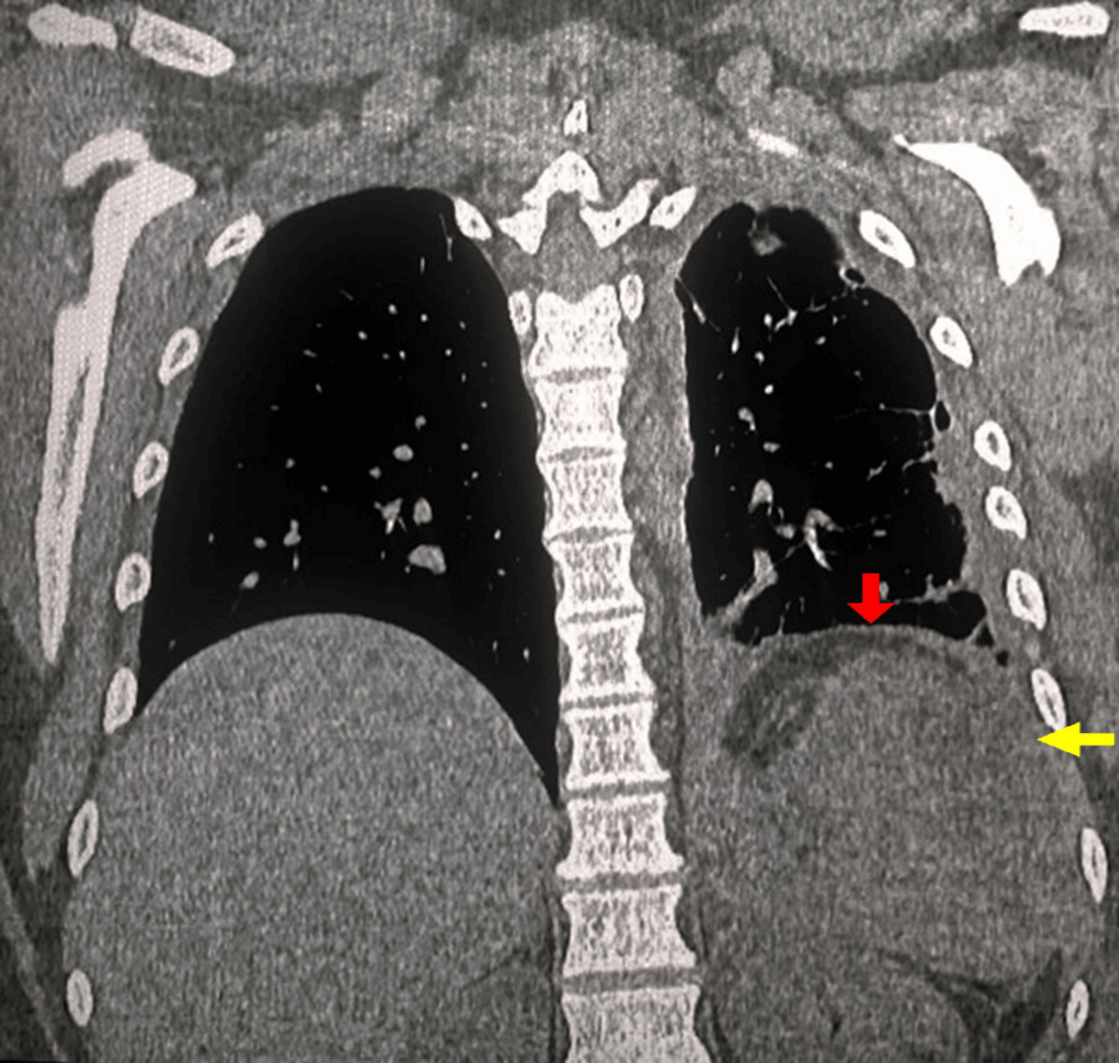
CT taken two weeks postoperatively demonstrated that the splenic hematoma (yellow arrow) had decreased in size and the pleural effusion had resolved (red arrow).

Three days later, besides worsening abdominal pain, the patient complained of left-sided chest pain, fever, and progressive dyspnea. Upon chest examination, the left lower zone showed diminished breath sounds with dullness on percussion. Leukocyte counts showed an upward trend, while hemoglobin levels remained stable. Accordingly, a Cystofix catheter was placed for pleural drainage, and the fluid was confirmed to be exudative. However, due to inadequate drainage and increasing pleural effusion, it was subsequently replaced with a chest tube. A follow-up chest-abdominal CT scan revealed a further increase in the left-sided pleural effusion with radiological features suggestive of organization, accompanied by enlargement of the splenic hematoma. (Figure 2). Consequently, a diagnostic and therapeutic thoracoscopy for pulmonary decortication was determined after the case was reviewed by the thoracic surgery team. Inevitably, thoracoscopy has been converted to thoracotomy owing to dense pulmonary adhesions. The splenic hematoma was successfully managed with percutaneous, ultrasound-guided drainage. A follow-up chest-abdominal CT performed one week later demonstrated resolution of the pleural effusion and a reduction in the size of the splenic hematoma (Figure 3). The patient was discharged on the 10th day after surgery.

## Discussion

Metabolic and bariatric surgeries have proved their efficacy as successful interventions for obesity and its associated disorders. However, the advancing prevalence of sleeve gastrectomy parallels the increasing chance of the challenging complication. While common complications associated with sleeve gastrectomy have been discussed thoroughly in the literature, there remains a paucity of reports highlighting rare complications. Pleural effusion secondary to splenic pathology is itself rare, with only isolated case reports describing splenic hematoma as a cause of exudative pleural effusion [7]. In the context of laparoscopic sleeve gastrectomy, pleural effusion has been reported but is typically attributed to other etiologies such as gastric leak, gastropleural fistula, or postoperative hemothorax rather than splenic hematoma [8]. To the best of our knowledge, reports specifically describing pleural effusion secondary to splenic hematoma following sleeve gastrectomy are extremely limited in the literature.

The mechanism underlying splenic hematoma after sleeve gastrectomy remains poorly defined in the literature. Several theories have been proposed, including inadvertent injury during mobilization of the greater gastric curvature, division of the gastrosplenic ligament, and the use of energy devices during sealing and transection of the short gastric vessels or dissection of the gastric fundus [4]. An alternative theory suggests that a minor capsular tear that occurs intraoperatively allows the residual pneumoperitoneum to auto-dissect the splenic capsule from the pulp as a low-resistance pathway, thereby facilitating hematoma expansion [9].

Pleural effusion in the postoperative setting may develop because of thoracic or abdominal complications, or both, such as esophageal perforation or peritoneal collections secondary to gastric or anastomotic leaks [10]. Splenic hematoma is a rare but clinically significant cause of pleural effusion [7]. Although most reported cases have occurred in trauma patients, it remains uncertain whether the pleural effusion results directly from the splenic hematoma or from concomitant injuries sustained during the primary trauma [11]. The exact mechanism underlying pleural effusion secondary to splenic hematoma remains poorly understood. Several hypotheses have been proposed, the majority of which implicate disturbances in the lymphatic drainage system. Unidirectional transdiaphragmatic vessels descend to drain the posterior thorax and receive tributaries from both the diaphragm and the splenic mesentery. Accordingly, pleural effusion in this context may result from a combination of direct compression of the posterior lymphatics by the enlarged spleen and transudation of hemorrhagic splenic fluid into the pleural space, facilitated by increased permeability from peri-splenic inflammation [7].

Left-sided pleural effusion has been highlighted as a strong red flag for a leak along the staple line after sleeve gastrectomy, and contrast-enhanced CT plays a central role in differentiating among these causes [12]. Management of postoperative pleural effusion should focus on addressing the underlying cause and be individualized according to the severity, while balancing the use of nonoperative strategies to maintain splenic function whenever feasible.

## Conclusions

A splenic hematoma that occurs after sleeve gastrectomy is a rare, curious cause of postoperative left-sided pleural effusion. Clinicians should be aware of this potential association, particularly in patients presenting with unexplained postoperative respiratory symptoms, as prompt recognition may aid timely diagnosis and management, thus preventing adverse outcomes.
